# Supplementary material for: Increasing sensitivity of dryland vegetation greenness to precipitation due to rising atmospheric CO2
Source: Nat Commun. 2022 Aug 19;13:4875. doi: 10.1038/s41467-022-32631-3 (PMC9391480; doi:10.1038/s41467-022-32631-3)
Supplement: Supplementary file 1 — Supplementary Information [file 41467_2022_32631_MOESM1_ESM.pdf]

Supplementary Information for

Increasing sensitivity of dryland vegetation greenness to precipitation due to rising atmospheric CO_2

Yao Zhang[1,2,3*], Pierre Gentine[4], Xiangzhong Luo[5], Xu Lian[1,4], Yanlan Liu[6], Sha Zhou[7], Anna M. Michalak[8], Wu Sun[8], Joshua B. Fisher[9], Shilong Piao[1,10], Trevor F. Keenan[2,3*]

[1]Sino-French Institute for Earth System Science, College of Urban and Environmental Sciences, Peking University, Beijing, China

[2]Climate and Ecosystem Sciences Division, Lawrence Berkeley National Laboratory, Berkeley, CA, USA

[3]Department of Environmental Science, Policy and Management, UC Berkeley, Berkeley, CA, USA

[4]Department of Earth and Environmental Engineering, Columbia University, New York, NY, USA

[5]Department of Geography, National University of Singapore, Singapore

[6]School of Earth Sciences, The Ohio State University, Columbus, OH, USA

[7]State Key Laboratory of Earth Surface Processes and Resources Ecology, Faculty of Geographical Science, Beijing Normal University, Beijing, China

[8]Department of Global Ecology, Carnegie Institution for Science, Stanford, CA, USA

[9]Schmid College of Science and Technology, Chapman University, Orange, CA, USA

[10]State Key Laboratory of Tibetan Plateau Earth System, Resources and Environment, Institute of Tibetan Plateau Research, Chinese Academy of Sciences, Beijing, China

[*]Corresponding author: zhangyao@pku.edu.cn; trevorkeenan@berkeley.edu

The SI contains Supplementary Discussion, 3 Supplementary Methods, 3 Supplementary Tables and 21 Supplementary Figures

Supplementary Discussion

We used different methods and datasets to test the robustness of θ_{prec} estimated from DLM. The spatial patterns of θ_{prec} trend calculated from multivariate linear regression (see Supplementary Methods 2) at both monthly and annual scales is consistent with DLM estimates (Supplementary Figure 3, 4). Both DLM and MLR methods show very good model performance, suggesting the sensitivity estimates are robust (Supplementary Figure 5). Using long-term leaf area index (LAI) dataset or other satellite NDVI products (see Methods), we also find a very similar pattern, which further supports our findings (Supplementary Figure 6, 7). The contrasting trends of θ_{prec} between drylands and non-drylands are robust regardless of the methods used, the precipitation datasets or vegetation indicators used (Supplementary Figure 8). The contrasting trends are mostly contributed by the sensitivity during the wet season when precipitation variability is large and more robust estimates are possible (Supplementary Figure 9). Our analysis of the θ_{prec} time-series also confirmed these results, with 7 out of 8 continents exhibiting greater trend values of θ_{prec} for drylands than non-drylands (Supplementary Figure 10).

Africa exhibits a significant increase in precipitation during the past decades. Precipitation trend directly affects the ecosystem water availability and consequently changes the vegetation sensitivity to precipitation. We further tested whether the observed contrasting trends of θ_{prec} is caused by precipitation trend in Africa. Using results from both DLM and MLR, we found that whether or not Africa is included does not affect our conclusion. Furthermore, much of the decreases in $\sigma NDVI_{prec}$ shown in Fig. 2 are contributed by drylands in Africa. If we exclude Africa from the analysis, the precipitation variability in drylands still decreases, while $\sigma NDVI_{prec}$ increases, suggesting a strong positive trend of θ_{prec} for the dryland (Supplementary Figure 11). In contrast, $\sigma NDVI_{prec}$ shows a greater reduction (-0.58% yr^{-1}) than the precipitation variability (-0.31% yr^{-1}) in non-drylands, suggesting that θ_{prec} decreased in non-drylands during this period (Fig. 2c).

Supplementary Method 1. Dynamic linear model

A DLM can be expressed as a combination of an observation equation (Eq. 1a) and a state evolution equation (Eq. 1b):

$$y_t = \mathbf{F}_t^T \boldsymbol{\theta}_t + v_t \tag{1a}$$

$$\boldsymbol{\theta}_t = G\boldsymbol{\theta}_{t-1} + \mathbf{w}_t \tag{1b}$$

where y_t is the observed NDVI at each month t after removing the mean. The first equation describes a linear model to predict y_t through observations ($\mathbf{F}_t$) and corresponding coefficients ($\boldsymbol{\theta}_t$). $\mathbf{F}_t$ is a vector consisting of three components, corresponding to the trend ($\mathbf{F}_{trend} = [1,0]$), seasonal ($\mathbf{F}_{seas} = [1,0,1,0,1,0]$), and a regression component ($\mathbf{F}_{reg,t} = [\delta Temp_t, \delta Cloud_{t,}, \delta Prec_{t,t-1}, \delta NDVI_{t-1},]$). $\delta Temp$, $\delta Cloud$, $\delta Prec$ represent the de-seasonalized detrended anomalies for temperature, cloud cover (a proxy of radiation), and precipitation, respectively. The subscript indicates the period when these anomalies were calculated. $\boldsymbol{\theta}_t$ is the state vector at time t, which also consists of three components: coefficients representing local mean and trend, coefficients representing seasonal dynamics, and regression coefficients for the environmental factors and lag-1 autocorrelation. v_t is the state evolution noise at time t assuming it has Gaussian noise with a zero mean. The second equation describes the evolution of the regression coefficients, where G is the state evolution matrix, which is diagonal connected by three small matrices also corresponding to the trend, seasonal and regression components. $\mathbf{w}_t$ is the state evolution noise at time t, following a zero mean multivariate Gaussian distribution.

Starting with non-informative $\boldsymbol{\theta}$ values at the first timestep, together with an $\mathbf{F}$ vector consisting of climate and lag-1 autocorrelation factors, the NDVI prior at timestep 1 can be predicted by Eq. (1a). We then used a Kalman filtering method to obtain a posterior estimate of y_1. The difference between the prior and posterior estimates of y_1 is backpropagated using Eq. (1a) so that posterior estimates of $\boldsymbol{\theta}_1$ can be obtained. The posterior of $\boldsymbol{\theta}_1$ is then used to predict *a priori* of $\boldsymbol{\theta}_2$ through Eq. (1b). This process is repeated for each timestep until a posterior time series of $\boldsymbol{\theta}$ is obtained. In this study, we specifically focused on the time series of the coefficient for precipitation, θ_{prec}, from which its mean and trend can be calculated for each pixel.

Supplementary Method 2. Multivariate linear regression

In addition to the DLM, we also used a multivariate regression to calculate the precipitation sensitivity and its trend. The entire study period was split into two halves, i.e., from 1981 to 1998 and from 1999 to 2015, and we compared the sensitivity changes between the two periods. We use multivariate linear regressions to estimate the sensitivity for precipitation for both periods. To understand the effect of different lengths of previous precipitation calculation, we tested different combinations and the model with best performance were used. The regression coefficient for precipitation is considered as the precipitation sensitivity (θ_{prec}). We also tested the selection of different breakpoints for the two periods (e.g., 1998,1999,2000), but this had a limited effect on the pattern we found (Supplementary Figure 3). The mean θ_{prec} is calculated as the average of θ_{prec} for both periods, and the trend is calculated as the difference between the two and adjusted to the annual value. In addition to this sensitivity calculation at the monthly scale, we also calculated the sensitivity at the annual scale, i.e., annual averages of NDVI and climate variables are used for the multivariate regression. To avoid splitting the growing season, each year starts from July and ends in June for the Southern hemisphere.

Supplementary Method 3. Minimalistic hydrological model

At the daily timescale, changes in the total soil water are a balance of input (precipitation, P) minus the losses to runoff (including leakage, Q), interception (E_I), transpiration (E_T), and soil evaporation (E_S):

$$nZ_r \frac{ds}{dt} = P[t] - E_s[s(t)] - E_T[s(t)] - E_I[t] - Q[s(t), t] \quad (2)$$

where s, Z_r and n are moisture content, vegetation rooting depth, and porosity of soil, respectively. The variables on the right-hand side are functions of time (t), soil moisture ($s(t)$) or both. This model is evaluated at annual scale to understand the hydrological partitioning under steady-state conditions, through which $\frac{\partial E_T}{\partial P}$ can be further calculated. The trend in precipitation sensitivity at annual scale is very similar to the one we show at monthly scale (Supplementary Figure 3, 4), and therefore, is helpful for understanding responses to CO_2. It should be noted that this model assumes a steady state for the equation derivation. Although strong seasonality in rainfall and temperature can alter the contribution of each factor, these effects are small and not considered here.

The model first assumes 15% of precipitation is intercepted by the vegetation canopy, following predictions from a series of Earth system models[1]. We also tested other values and found this assumption does not directly affect our results afterwards (Supplementary Figure 18). Interception contributes directly to the total latent heat, with remaining latent heat fluxes fulfilled by evapotranspiration from soil and vegetation ($E_T + E_S$), which decrease linearly from the maximum potential evapotranspiration (E_P) when soil is at field capacity (s_{fc}) to 0 when at the hygroscopic value (s_h). Below the wilting point of soil (s_w), all evapotranspiration losses are through surface evaporation, because vegetation cannot extract water during this period. Above this point, transpiration is proportional to the available soil water. This suggests that the soil moisture dynamics are key to the water partitioning to transpiration and evaporation. We defined a "relative" soil moisture (x) by linearly scaling the soil moisture between the hygroscopic value and field capacity, $x = (s - s_h)/(s_{fc} - s_h)$, and similarly, a relative wilting point $\omega = (s_w - s_h)/(s_{fc} - s_h)$. The soil moisture dynamics are affected by precipitation (characterized by frequency, λ, and depth, α), root zone soil water storage ($w_0 = (s_{fc} - s_h)nZ_r$), as well as the dryness index ($\phi = E_P/P$, inverse of aridity index). For calculation, we also defined a ratio of soil water storage to average rainfall depth ($\gamma = w_0/\alpha$). Since interception will alter the rainfall frequency and depth arriving at the soil surface, and consequently the dryness index calculation, we followed a previous study[2] and recalculate the abovementioned factors after interception, denoted with a prime symbol (′): $\lambda' = \lambda e^{-\delta}$, $\alpha' = (1 - \delta)\alpha$, $P' = (1 - \delta)e^{-\delta}P$, $E_P{}' = E_P - E_I$, $\phi' = (\phi - 1 + (1 - \delta)e^{-\delta})/((1 - \delta)e^{-\delta})$, and $\gamma' = \gamma/(1 - \delta)$. where δ represents the ratio of canopy water storage (Δ) to average rainfall intensity: $\delta = \Delta/\alpha$. The probability distribution for the relative soil moisture is:

$$p(x) = \frac{x^{\frac{\gamma'}{\phi'}-1} e^{-\gamma' x} \gamma'^{\frac{\gamma'}{\phi'}}}{\Gamma\left(\frac{\gamma'}{\phi'}\right) - \Gamma\left(\frac{\gamma'}{\phi'}, \gamma'\right)} \tag{3}$$

Because E_S is a constant above the wilting point, the fraction of transpiration to evapotranspiration from soil, $f = E_T/(E_T + E_S)$, equals $(x - \omega)/x$ when soil moisture is above the wilting point. The expectation of f can be calculated as:

$$f = \int_{\omega}^{1} p(x)\left(\frac{x - \omega}{x}\right) dx$$

$$= \frac{\left[\Gamma\left(\frac{\gamma'}{\phi'}, \omega\gamma'\right) - \Gamma\left(\frac{\gamma'}{\phi'}, \gamma'\right)\right] - \omega\gamma'\left[\Gamma\left(\frac{\gamma'}{\phi'} - 1, \omega\gamma'\right) - \Gamma\left(\frac{\gamma'}{\phi'} - 1, \gamma'\right)\right]}{\Gamma\left(\frac{\gamma'}{\phi'}\right) - \Gamma\left(\frac{\gamma'}{\phi'}, \gamma'\right)} \tag{4}$$

where $\Gamma(\cdot)$ and $\Gamma(\cdot,\cdot)$ represents complete and upper incomplete gamma functions, respectively. γ' and ϕ' are modified for soil surface conditions after considering canopy interception.

The surface evapotranspiration from soil is scaled by average effective soil moisture ($\bar{x}$) from potential evapotranspiration $E_P{}'$, similarly, the prime superscript indicates this is E_P adjust for canopy interception

$$\frac{E_T + E_S}{P} = \frac{\bar{x}E_P{}'}{P'}\frac{P'}{P} = \bar{x}\phi'\rho \tag{5}$$

where ρ indicate the average throughfall fraction (85%). And the average effective soil moisture ($\bar{x}$) can be calculated as:

$$\bar{x} = \frac{1}{\phi'} - \frac{\gamma'^{\frac{\gamma'}{\phi'}-1} e^{-\gamma'}}{\Gamma\left(\frac{\gamma'}{\phi'}\right) - \Gamma\left(\frac{\gamma'}{\phi'}, \gamma'\right)} \tag{6}$$

CO_2 affects E_T through both direct effect of reducing stomatal conductance (g_s) and indirect effect of increasing LAI. To consider these two effects in the minimalistic model, we used a scaling factor κ to represent the proportional stomatal closure due to CO_2 and a scaling factor ζ to represent the proportional LAI increase due to CO_2. We used a widely used stomatal conductance model[3] to calculate this κ value:

$$\kappa = \frac{g_0 + (1 + \frac{g_1}{\sqrt{D}})\frac{A + \Delta A}{C_a + \Delta C_a}}{g_0 + (1 + \frac{g_1}{\sqrt{D}})\frac{A}{C_a}} \approx \frac{\frac{A + \Delta A}{A}}{\frac{C_a + \Delta C_a}{C_a}} = \frac{1 + \beta_{CO_2}\frac{\Delta C_a}{C_a}}{1 + \frac{\Delta C_a}{C_a}} \tag{7}$$

where g_0, g_1 are empirical parameters representing the minimum stomatal conductance and the sensitivity of stomatal conductance to the vapor pressure deficit (VPD or D). C_a and A represent the ambient CO_2 concentration and photosynthesis, respectively. ΔC_a and ΔA indicate the change in ambient CO_2 and resultant increase in photosynthesis. β_{CO_2} is the CO_2 sensitivity calculated as $\frac{\partial A/A}{\partial C_a/C_a}$. Using C_a=354ppm (average for 1981-1998), ΔC_a=30ppm, we estimated κ_{C3} as 0.969 for C3 plants with β_{CO_2}=60% (ref. [4]), while for C4 plants, we estimated this κ_{C4} as 0.922 with

β_{CO_2}=0%. This indicates the average g_s in the second half of the study period (1999-2015) will decrease to 96.9% (for C3 plants) or 92.2% (for C4 plants) of its average value in the first half (1981-1998), and so will transpiration if changes of climatic factors are not considered.

To estimate ζ, we used an hypothetical model from a previous study[5]. ζ shows a strong dependency along the resource availability index, with higher values in the low resource regions and lower values (close to zero) in the high resource regions. This hypothetical model is well supported by the free air CO_2 enrichment (FACE) experiments throughout the globe. For our study region, water is the primary limiting resource, we therefore used the dryness index instead of the resource availability index in our study. ζ is assumed to have a nonlinear relationship with the dryness index (ϕ):

$$\zeta = 1 + b(\frac{1}{1 + e^{a/\phi}}) \quad (8)$$

where b is the coefficient that determines the maximum LAI increase at the dry end. ζ is suggested to increase by ~19% for the second half (1999-2015) as compared to the first half (1981-1998), based on the estimates from arid ecosystems in Australia[6]. We therefore set b as 0.38 so that ζ is in the range of (1, 1.19]. It should be noted that this value is much greater than the stomatal reduction at the dry end, likely due to an increase in the fraction of precipitation being partitioned into transpiration. a is the coefficient determining the slope of the effect along ϕ. We set it to 4 in this study so that the shape of the response is similar to a previous studies[7]. Using other a and b values does not qualitatively affect our results (Supplementary Figure 21).

Combining Eq. (4-8), we can get

$$\frac{E_T}{P} = f\bar{x}\phi'\rho\kappa\zeta = g(\phi) \quad (9)$$

Although this minimalistic model is simplified, it can very well capture the evapotranspiration fraction ($\frac{E_T+E_S+E_I}{P}$) predicted by the Budyko framework[8]. The fraction of transpiration to precipitation also matches the observations and model simulations at the global scale. By adding the effect of stomatal conductance (κ) and LAI (ζ), this model is also capable of responding to CO_2 both directly and indirectly.

From Eq. (9), we can calculate the partial derivative of E_T to P:

$$\frac{\partial E_T}{\partial P} = g(\phi) - \frac{\partial g(\phi)}{\partial \phi}\phi \tag{10}$$

The partial derivative of $g(\phi)$ to ϕ is difficult to calculate analytically, we therefore calculated it numerically. To do this, we used sandy loam soil and global average rooting depth of 67.8 cm, with global average rainfall depth of 7.4mm. These parameter settings have been used by previous studies and shown rather good performance. We showed that different values used here does not directly affect our results.

Supplementary Table 1. MsTMIP simulation scenarios used in this study.

Scenarios	Climate Forcing	Land-Use History	Atmospheric CO_2	Nitrogen Deposition
SG1	CRU+NCEP v6	Constant	Constant	Constant
SG2	CRU+NCEP v6	Time-varying	Constant	Constant
SG3	CRU+NCEP v6	Time-varying	Time-varying	Constant
BG1	CRU+NCEP v6	Time-varying	Time-varying	Time-varying

The CRU-NCEP V6 is at 0.5° spatial and 6-hourly temporal resolution, factors including incoming longwave/shortwave radiation, air temperature and humidity, pressure, wind speed, precipitation are considered. Land use history is from Hurtt's+SYNMAP. CO_2 data is from Enhanced GlobalView. Nitrogen deposition data is from Enhanced Dentener. For detailed information, see Wei et al., 2014 (ref. [9])

Supplementary Table 2. MsTMIP models and simulations used in this study.

Model	Simulation scenarios	Nitrogen cycle	Reference
BIOME-BGC	SG1, BG1	Yes	[10]
CLASS-CTEM-N	SG1, SG2, SG3, BG1	Yes	[11]
CLM4	SG1, SG2, SG3, BG1	Yes	[12]
CLM4VIC	SG1, SG2, SG3, BG1	Yes	[13]
GTEC	SG1, SG2, SG3	No	[14,15]
LPJ-wsl	SG1, SG2, SG3	No	[16]
ORCHIDEE-LSCE	SG1, SG2, SG3	No	[17]
SiBCASA	SG1, SG2, SG3	No	[18]
VEGAS2.1	SG1, SG2, SG3	No	[19]
VISIT	SG1, SG2, SG3	No	[20]

Supplementary Table 3. Parameters used in the minimalistic model

	Possible values	Note
Soil types	sand, sandy loam, loam, clay	Soil type determines the porosity (n), field capacity (s_{fc}), wilting point (s_w), hygroscopic value (s_h)
Rooting depth (Z_r, in mm)	672, 370, 3140	These values represent the global mean, lower (shrubland) and upper (evergreen broadleaf forest) bound from Yang et al. 2016 (ref. [21])
Rainfall depth (α, in mm)	7.4, 4, 16	These values represent the global land mean, lower and upper bound estimates from Goods et al. (ref. [2])
Canopy storage (Δ, in mm)	0.588, 0.385, 0.799	These values correspond to three levels of interceptions when they take 15%, 10%, and 20% of total precipitation (assuming α=7.4).

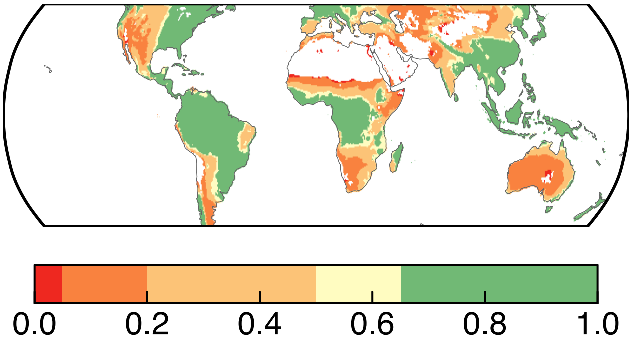

Supplementary Figure 1. The map of the aridity index (P/PET) for the study region. Colors from red to green correspond to hyper-arid, arid, semi-arid, dry sub-humid, and humid, respectively. Dryland corresponds to areas with an aridity index smaller than 0.65 (i.e., red, orange and yellow color). White area represents barren land with no vegetation.

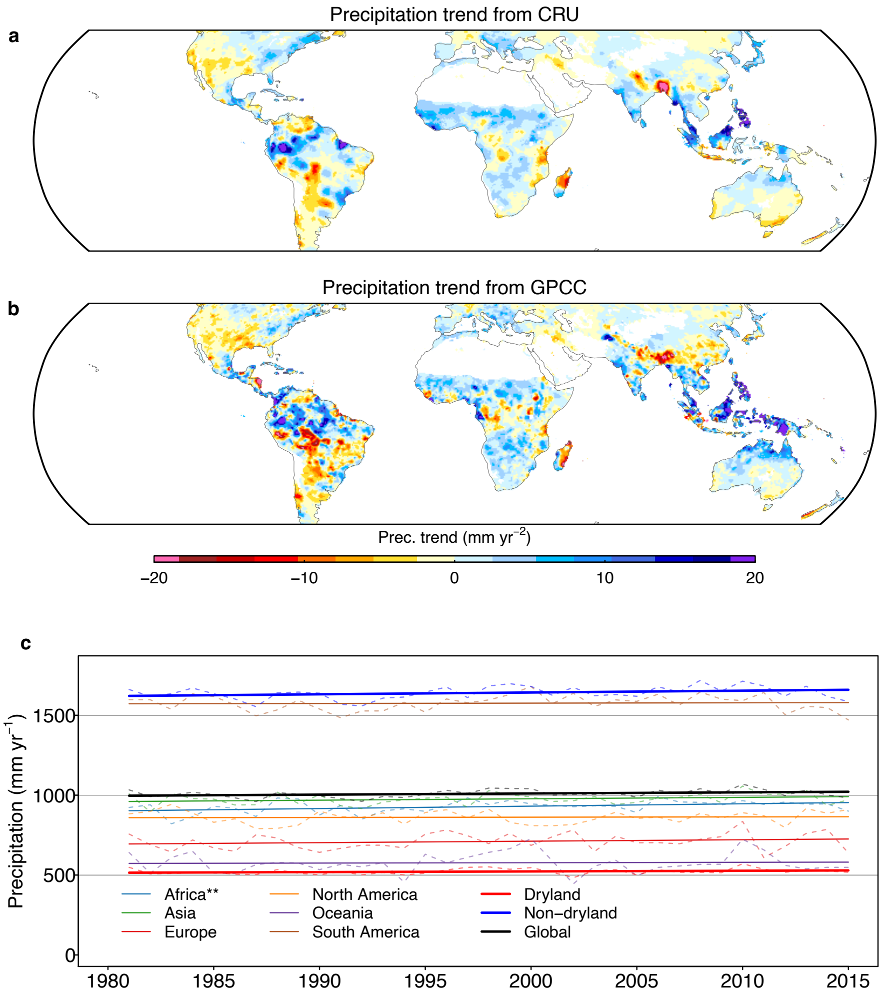

Supplementary Figure 2. Trend in precipitation during 1981-2015. **a,b** spatial patterns of precipitation trend from CRU and GPCC dataset. **c** Precipitation variabilities for the 6 continents within our study area (50°N-50°S). Dashed lines indicate the average precipitation for the vegetated area for each continent, and the solid lines indicates the linear regression. Only Africa shows a significant trend (Sen's slope=1.16 mm yr^{-2}, P=0.01 for CRU and Sen's slope=1.10 mm yr^{-2}, P=0.05 for GPCC).

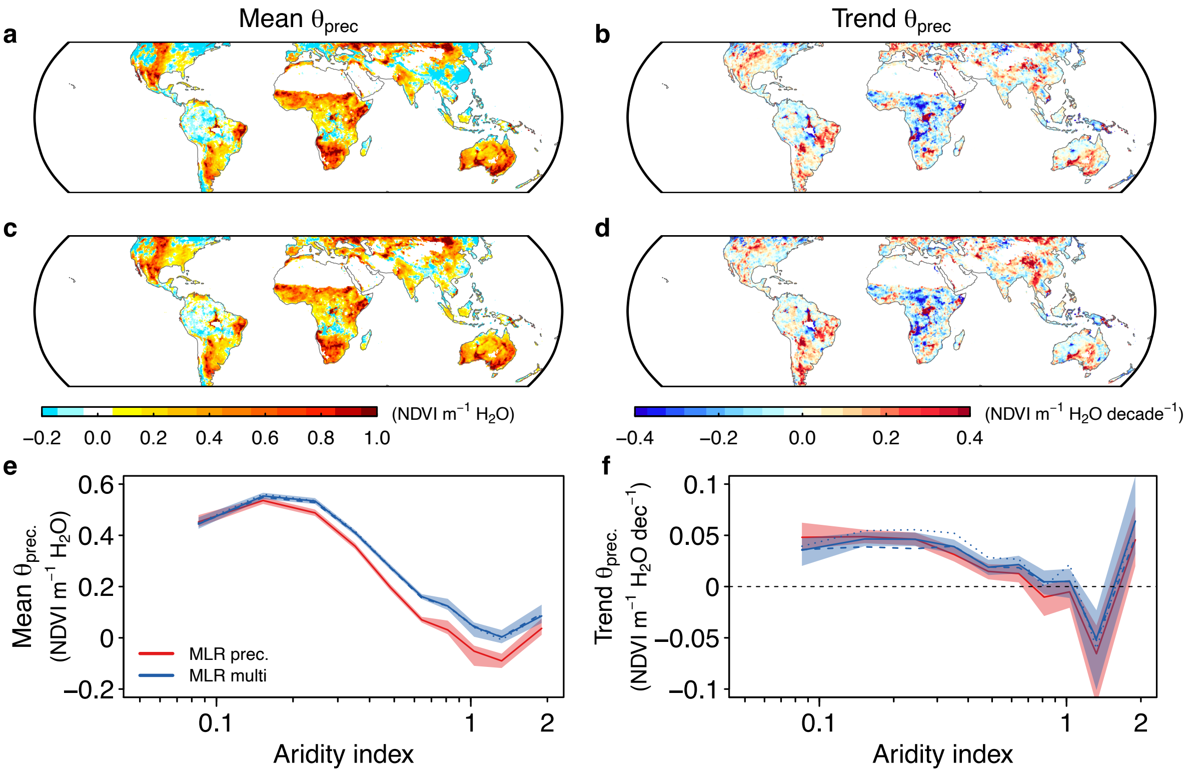

Supplementary Figure 3. Precipitation sensitivity using the multivariate linear regression. a and **b** are from models that only include lag-1 autocorrelation and precipitation. We use different periods to calculate the precipitation anomalies, and the one with best model performance is used for each pixel. **c** and **d** are from models that include additional current month temperature and cloud fraction. **e** and **f** indicate the mean and trend of precipitation sensitivity along the aridity index. The red and blue lines indicate the sensitivity calculated from the model that includes lag-1 autocorrelation and precipitation, or uses additional temperature, cloud fraction. Dash and dotted lines indicate mean and trend of θ_{prec} with a breakpoint of year 1997 and 1999, respectively. The lines indicate the median value and the shades indicate the 95% confidence interval calculated in each aridity bin through bootstrapping (n=5000).

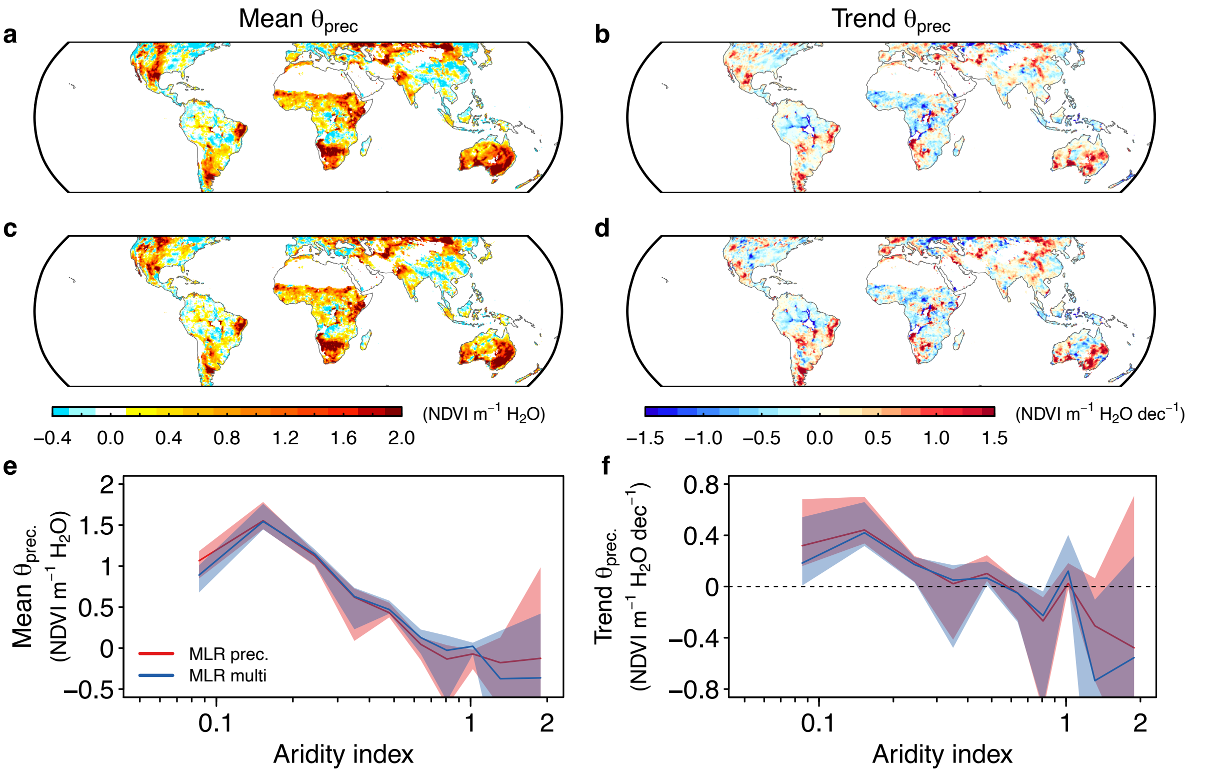

Supplementary Figure 4. Precipitation sensitivity obtained at annual scale. a and **b** are from models that only include lag-1 autocorrelation and precipitation. **c** and **d** are from models that include additional current month temperature and cloud fraction. Anomalies of annual averages of NDVI and climate variables are used in the regression. **e** and **f** show the mean precipitation sensitivity and trend of precipitation sensitivity, respectively. Shades indicate the 95% confidence interval calculated in each aridity bin through bootstrapping (n=5000).

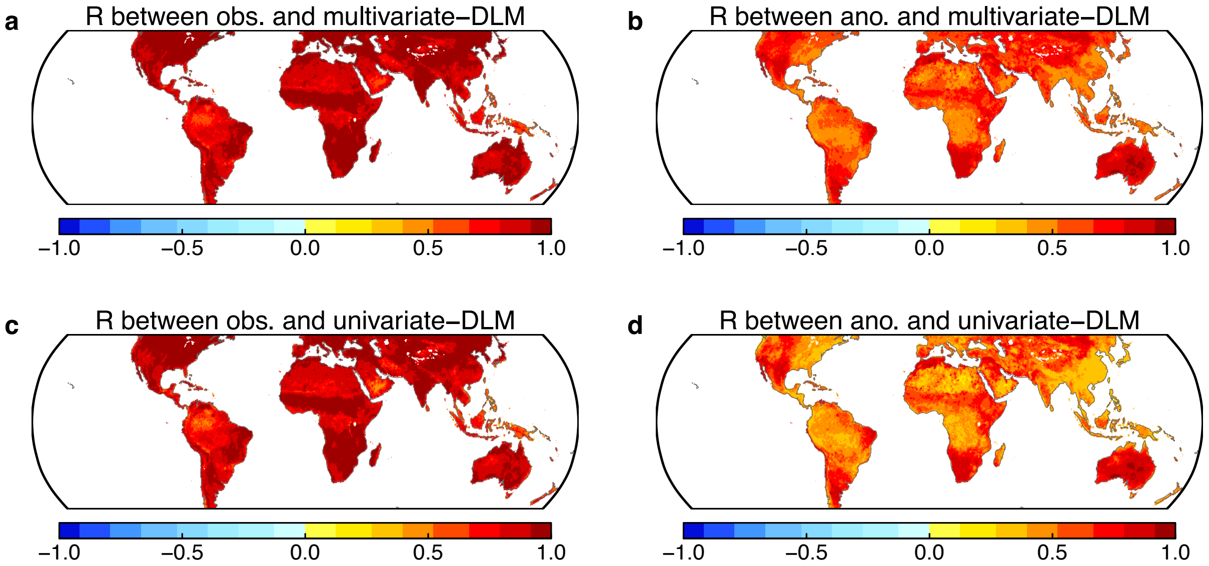

Supplementary Figure 5. performance of the models. Multivariate DLM including precipitation, temperature, cloud fraction and previous month VI (**a, b**). Univariate DLM only includes precipitation and previous month VI (**c, d**). Left column indicates the correlation coefficient (R) for model predicted value and observed NDVI. Right column indicates the R between de-seasonalized detrended anomalies observed and predicted by DLM.

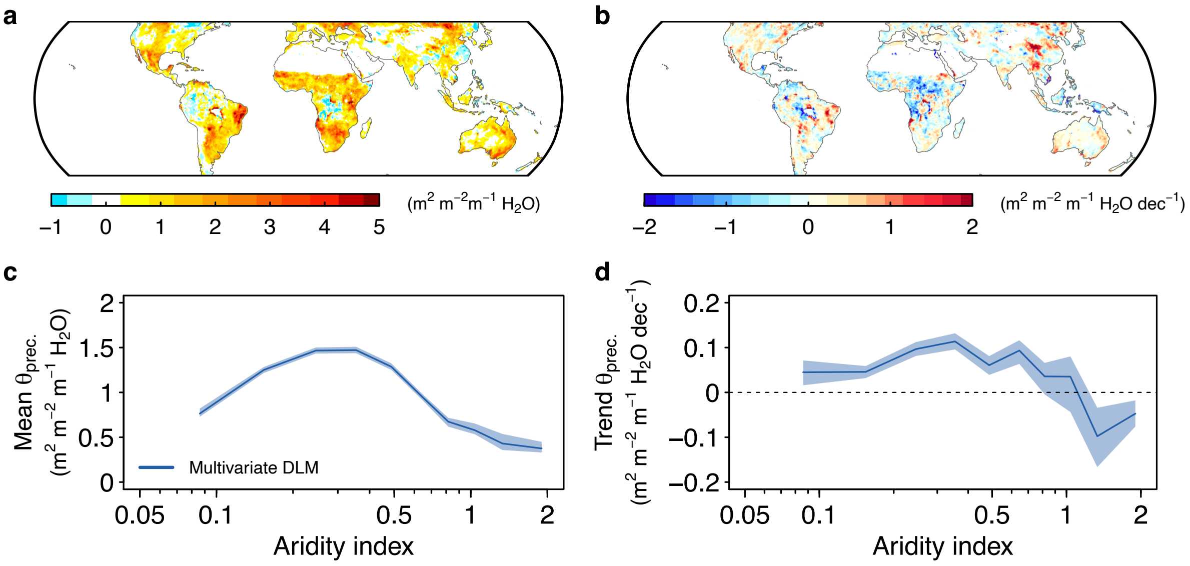

Supplementary Figure 6. Precipitation sensitivity calculated using GIMMS LAI 3g dataset. **a** and **b** are from multivariate DLM that includes lag-1 autocorrelation and three climate variables. **c** and **d** **e** and **f** show the mean precipitation sensitivity and trend of precipitation sensitivity, respectively. Shades indicate the 95% confidence interval calculated in each aridity bin through bootstrapping (n=5000).

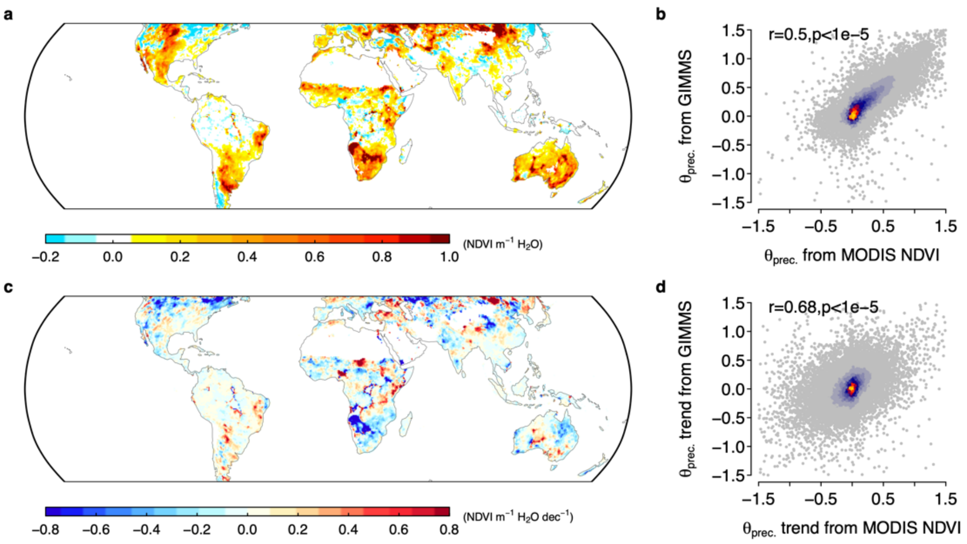

Supplementary Figure 7. Precipitation sensitivity calculated using MODIS NDVI dataset during 2001-2015. **a** and **c** are for the mean and trend of θ_{prec}, respectively. **b** and **d** show the comparison of the spatial patterns between mean θ_{prec} and trend in θ_{prec} from either MODIS (x-axis) or GIMMS 3g (y-axis) based on the one-sided t-test during the same period (2001-2015). Warmer color indicates higher density.

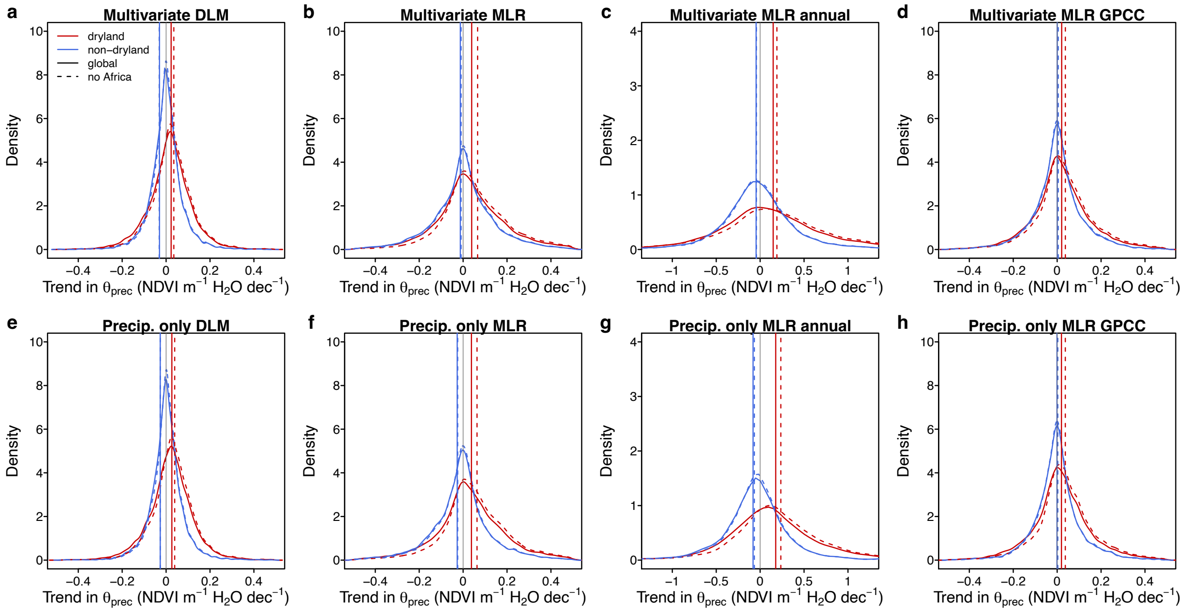

Supplementary Figure 8. Trend in precipitation sensitivity (θ_{prec}) over dryland and non-dryland. Trend in θ_{prec} estimated from dynamic linear models (DLM, **a,e**), multivariate linear regression (MLR, **b,f**), MLR at annual time step (**c,g**) and monthly MLR with GPCC precipitation (**d,h**). Upper panel considers additional climate variables in the regression (Eq. (2)) while lower panel considers precipitation anomalies as the only climate variable (Eq. (3)). Solid lines indicate the density for the entire low latitude regions, while dashed lines indicate the density with Africa removed. Vertical lines indicate the average value of the trend in θ_{prec} for either dryland or non-dryland. Dryland and non-dryland area separated by the aridity index of 0.65.

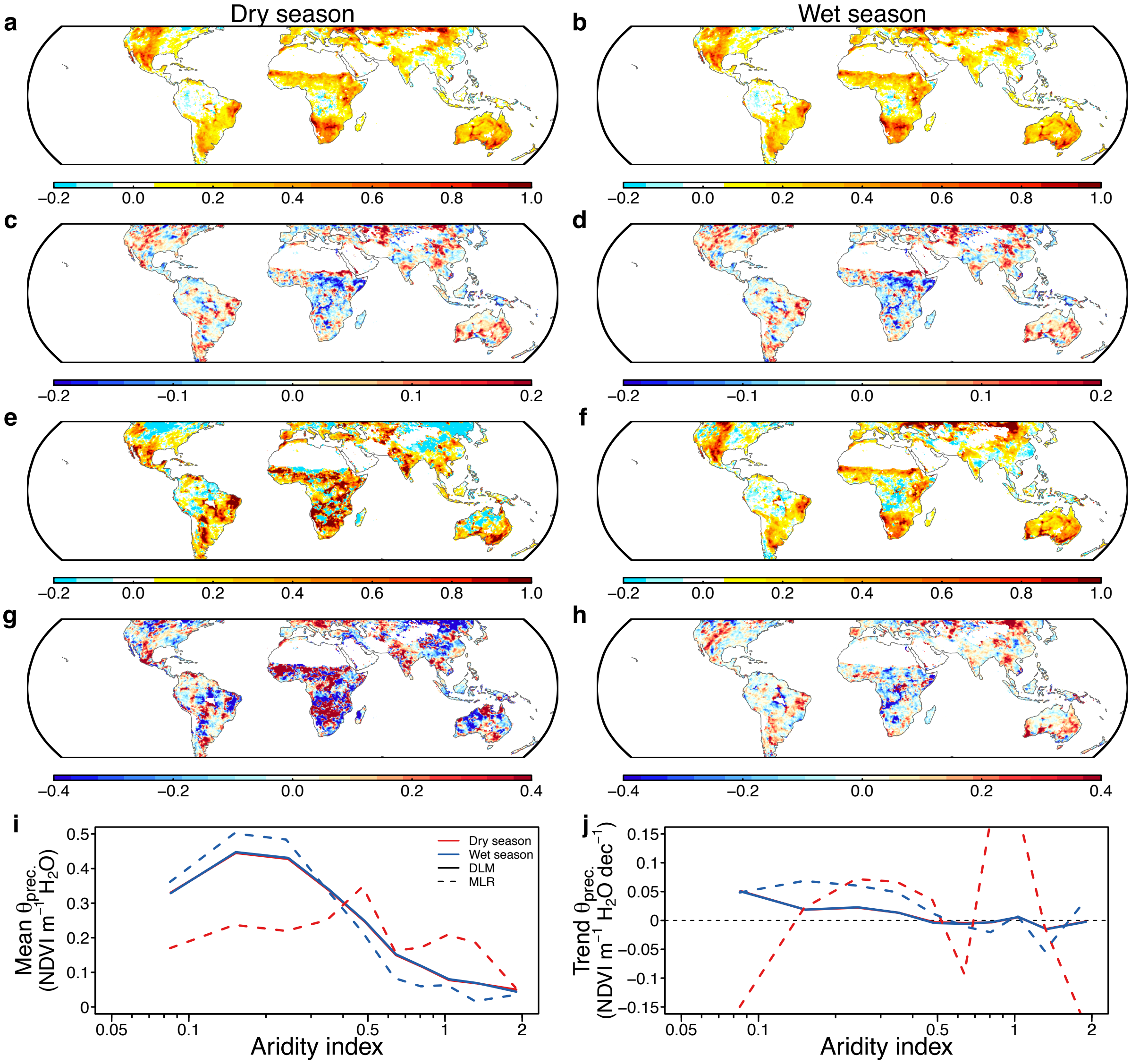

Supplementary Figure 9. Comparison of precipitation sensitivity for dry and wet season. The dry and wet seasons are defined as three consecutive months with smallest and greatest climatological precipitation. Maps in the left column shows the dry season and right column shows the wet season. **a-d** results from multivariate DLM; **e-h** results from MLR. **a, b, e, f** are for the mean sensitivity and **c, d, g, h** are for the trend in sensitivity. Both DLM and MLR use precipitation, cloudiness, and temperature as environmental drivers. mean (**i**) and trend (**j**) of sensitivity along the aridity index. For DLM, sensitivity is for either dry or wet season is calculated for each year and then used to get the mean and trend. For MLR, de-seasonalized NDVI and climate variables for dry and wet seasons are selected first and the precipitation sensitivity is then calculated for first and second half and for dry and wet season separately. Precipitation sensitivities show large variations during the dry season using MLR method, mostly due to limited precipitation variations and poor model performance.

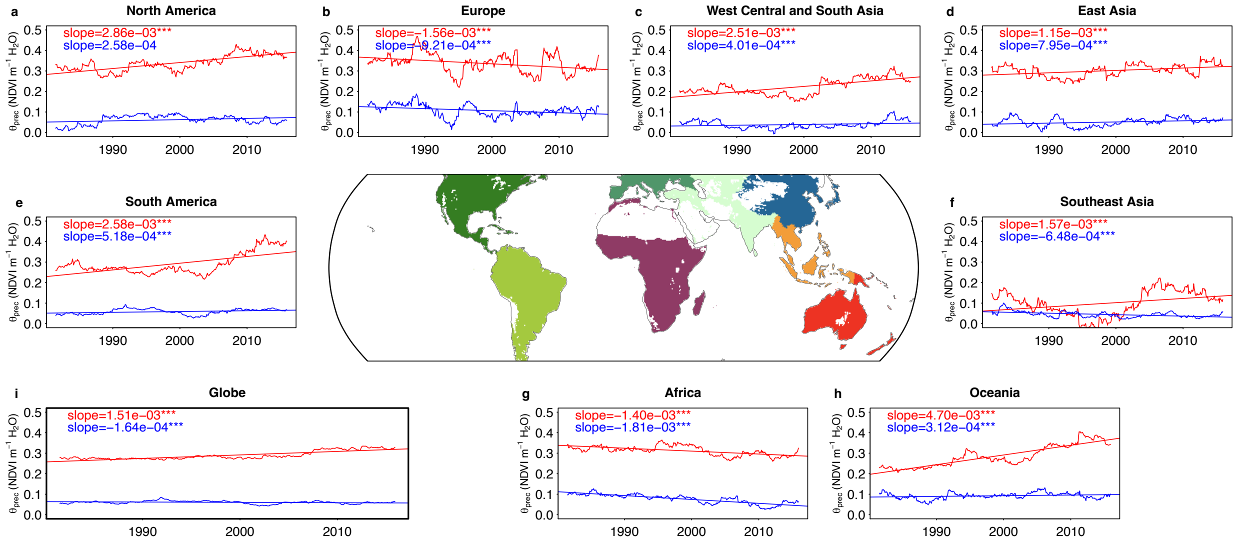

Supplementary Figure 10. Times series of vegetation sensitivity to precipitation for dry and wet regions for each continent. a-g median value of θ_{prec} for each continent. **i** median value for the entire globe. Red and blue lines indicate θ_{prec} for dryland and non-dryland, respectively. Slope values in the upper left corner of each subplot show the trend estimated from the Sen's slope estimator, with "***" indicate the trend is significant at P<0.001 based on the two-sided Mann Kendall test.

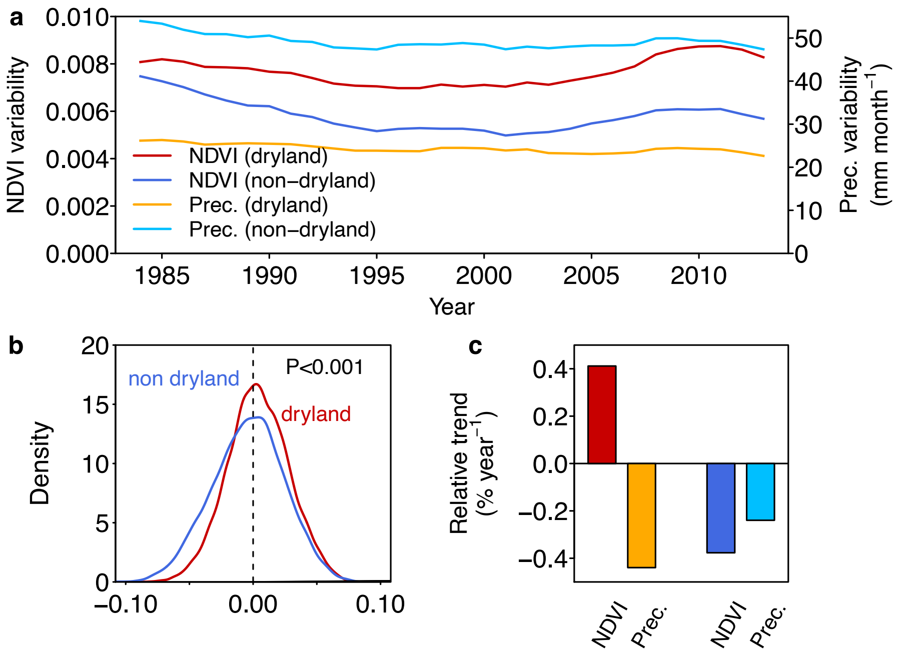

Supplementary Figure 11. Trend in variability of vegetation related to the precipitation. a Same as Fig. 2b, but for statistics without Africa. **b** Probability density of $\sigma NDVI_{prec}$ for dryland and non-dryland (with Africa excluded), the P value is obtained based on a one-sided t-test. **c** same as Fig. 2c, but for statistics without Africa.

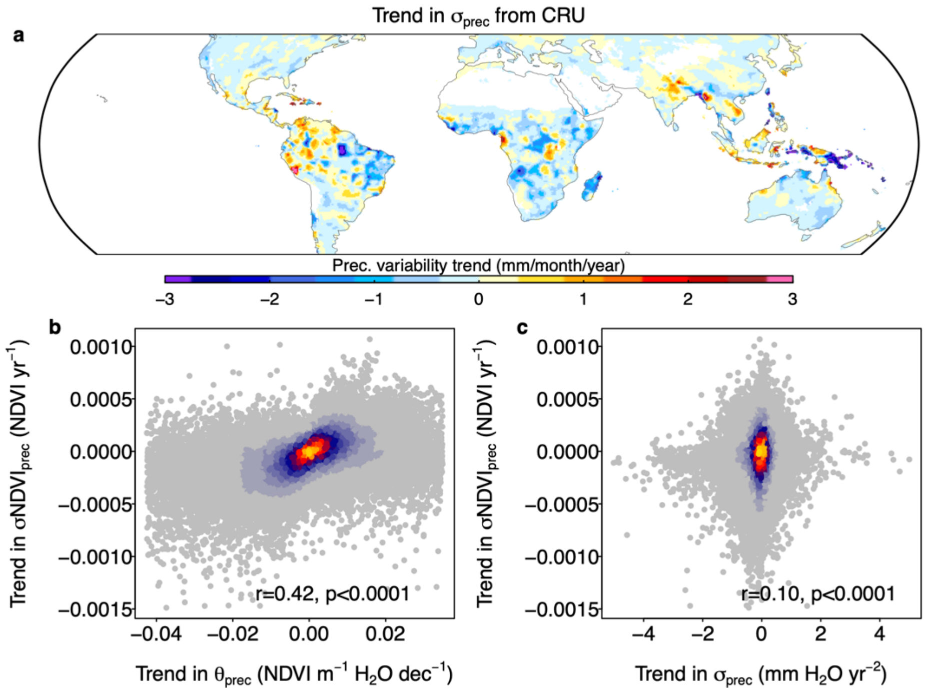

Supplementary Figure 12. Trend in precipitation variability (σ_{prec}) and its relationship with trend in NDVI variability ($\sigma NDVI_{prec}$). Trend in de-seasonalized detrended precipitation variability (σ_{prec}) during 1982-2015 from CRU TS 4.04 (**a**). The variability is calculated within a 5-year moving window and the trend is calculated using the non-parametric Sen's slope estimator. **b** comparison between the trend in NDVI variability associated with precipitation ($\sigma NDVI_{prec}$) with trend in θ_{prec}. **c** comparison between the trend in NDVI variability associated with precipitation ($\sigma NDVI_{prec}$) with trend in σ_{prec}. The correlations for (**b**) and (**c**) are evaluated using a one-sided t-test. Warmer color indicates higher density.

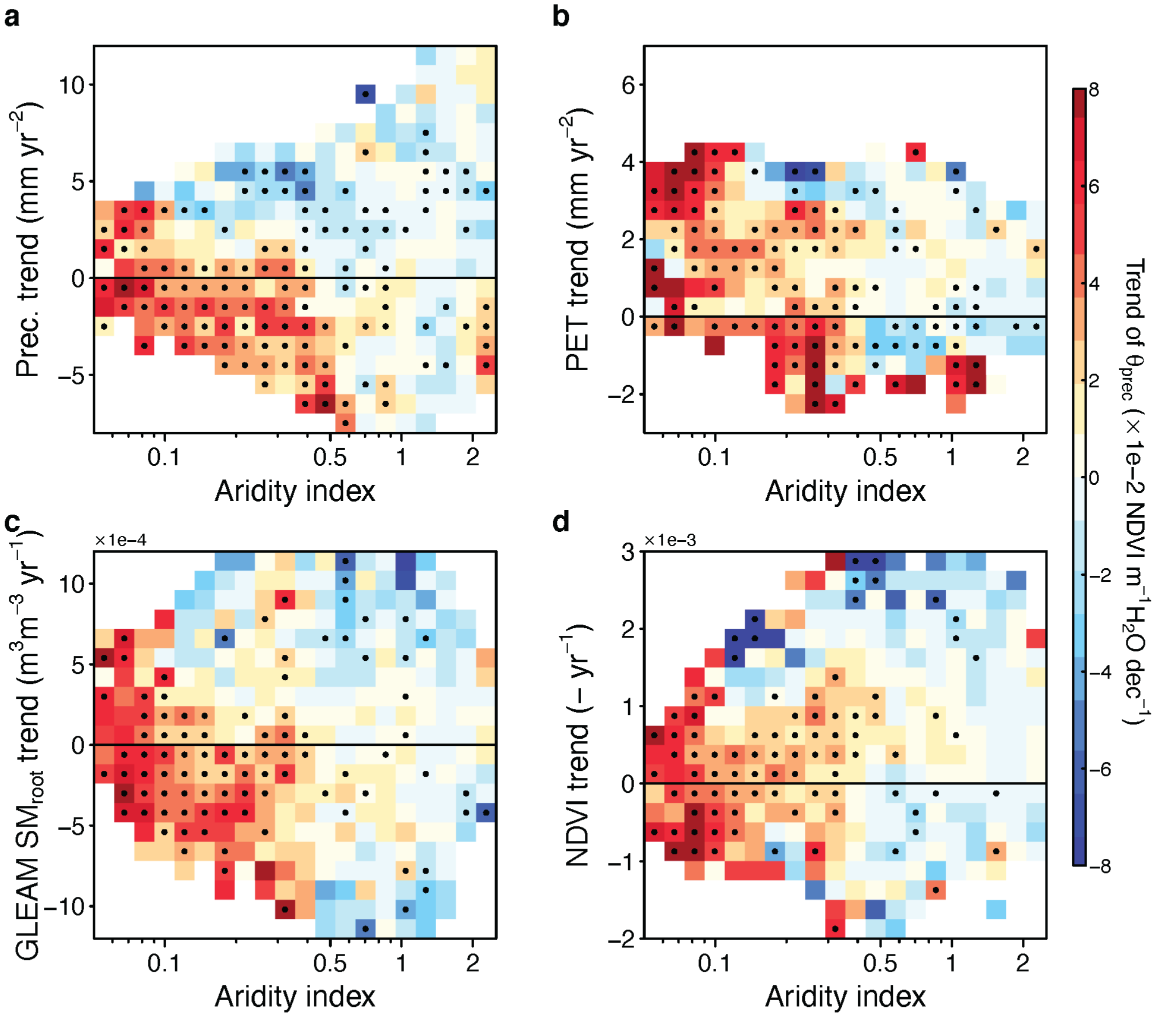

Supplementary Figure 13. Trend of precipitation sensitivity (θ_{prec}) along vegetation and climate trends. **a** trend of θ_{prec} along aridity index and precipitation trend, **b** trend of θ_{prec} along aridity index and potential evapotranspiration trend from CRU TS 4.05, **c** trend of θ_{prec} along aridity index and GLEAM root zone soil moisture trend, **d** trend of θ_{prec} along aridity index and mean annual GIMMS NDVI trend. Median value is shown for each grid and dots indicate trend of θ_{prec} is significant at P<0.05 based on the two-sided t-test.

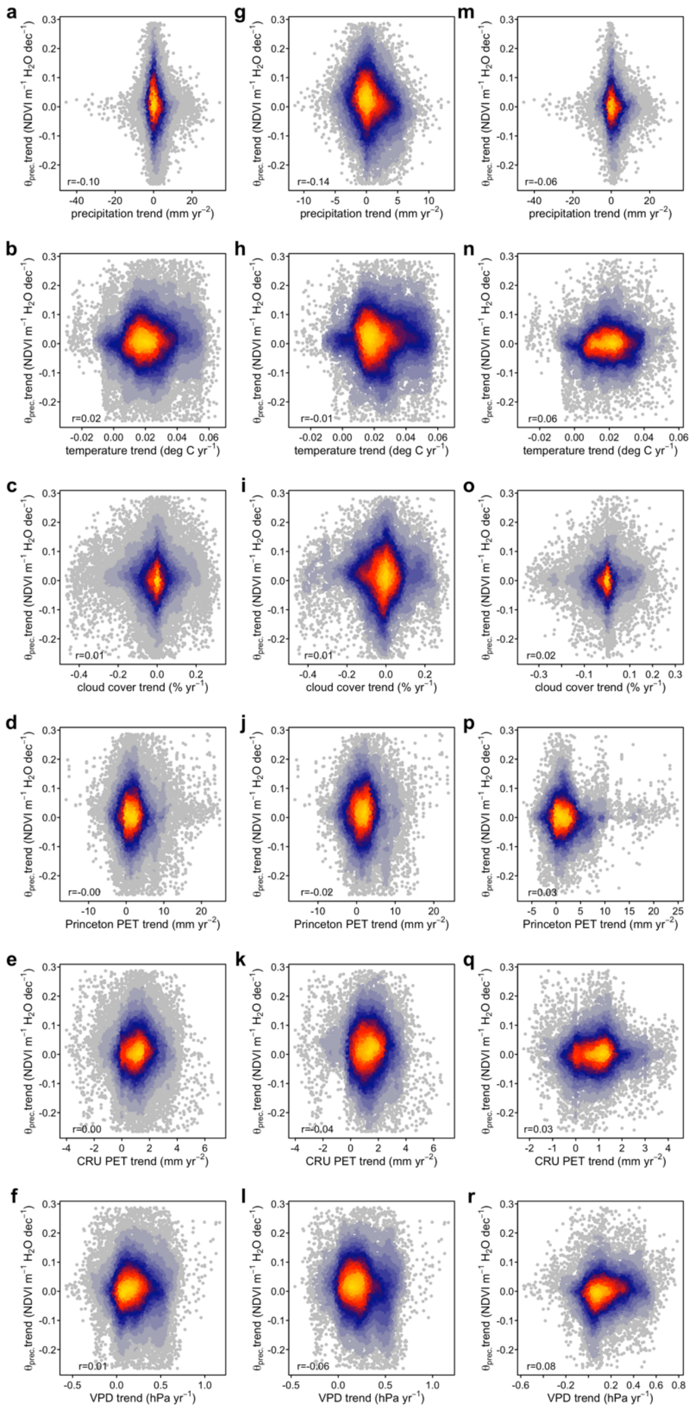

Supplementary Figure 14. Relationship between trend of precipitation sensitivity and trend in climate variables (precipitation, temperature, cloud cover, potential evapotranspiration, and vapor pressure deficit). The first column shows the correlation for all pixels (**a-f**), second column for the dryland pixels (**g-l**), third column for the non-dryland pixels (**m-r**). PET from both Princeton[22] and CRU TS 4.05 are used. Warmer color indicates higher density.

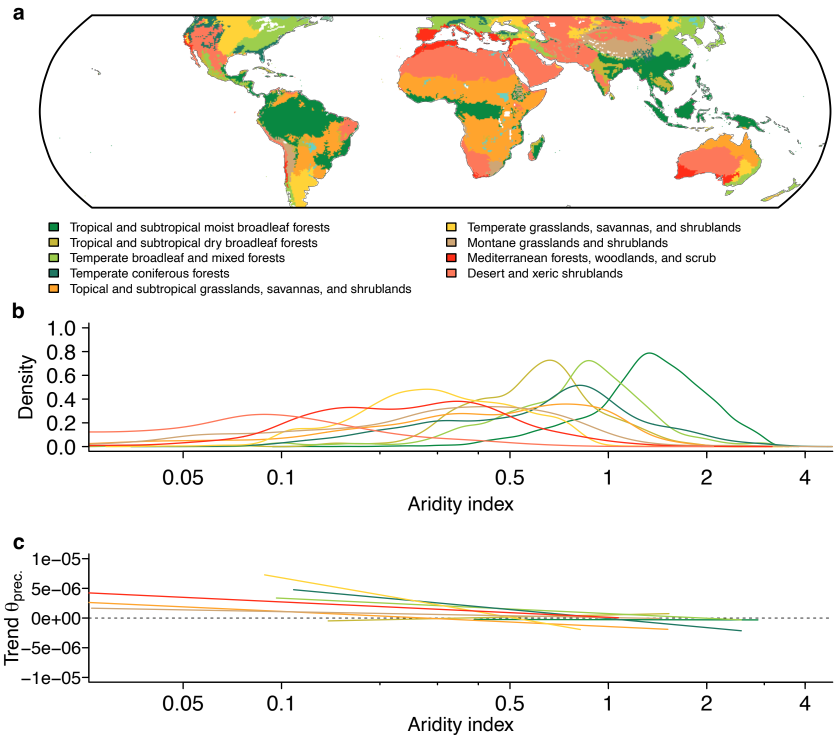

Supplementary Figure 15. Vegetation sensitivity to precipitation along aridity index for each biome types. a the Olson's biome map for our study region. **b** the distribution of biome along the aridity index. **c** relationship between θ_{prec} and aridity index within each biome type.

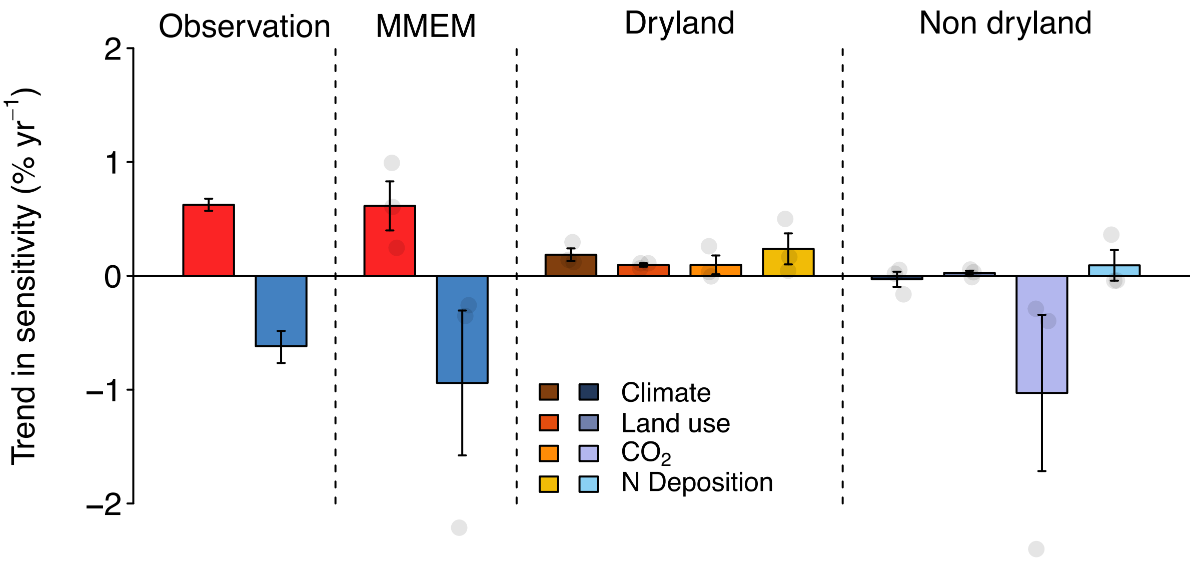

Supplementary Figure 16. Attribution of changes in precipitation sensitivity using MsTMIP model experiments. similar as Fig. 3, but only using three models (CLASS-CTEM-N, CLM4 and CLM4VIC) which have all simulation scenarios. The bars and error bars indicate the mean and standard error of the mean (SEM) of median trend across models, respectively. The sample sizes for each factor are: Observation 17601 and 14095 (for dryland and non-dryland, respectively); MMEM 3 and 3; Climate 3 and 3; Land use 3 and 3; CO_2 3 and 3; N Deposition 3 and 3.

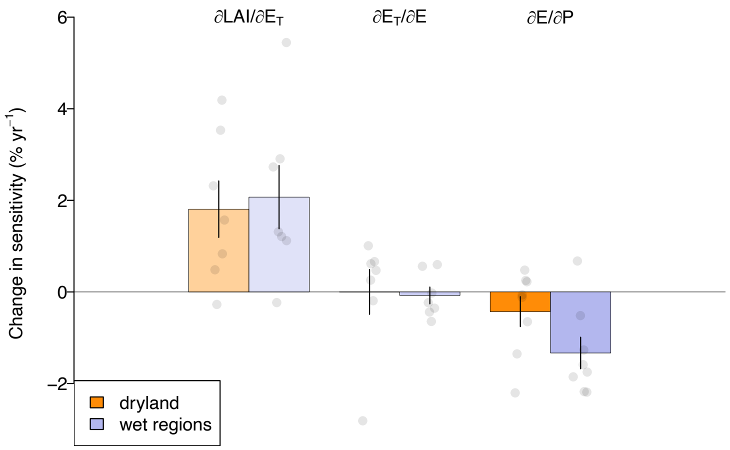

Supplementary Figure 17. Attribution of CO_2 effect on changes in precipitation sensitivity using MsTMIP model. The three groups from left to right (lighter to darker color) indicates the response of LAI to transpiration (E_T), E_T to evapotranspiration (E), and E to precipitation (P). Results were calculated as a multi-model ensemble mean. Due to the availability of the variables available, VEGAS is not used. The bars and error bars indicate the mean and standard error of the mean (SEM) of median trend across models, respectively. The sample sizes for each factor are: $\partial \mathrm{LAI}/\ \partial \mathrm{E_T}$ 7 and 7 (for dryland and non-dryland, respectively); $\partial \mathrm{LAI}/\ \partial \mathrm{E_T}$ 7 and 7; $\partial \mathrm{LAI}/\ \partial \mathrm{E_T}$ 8 and 8.

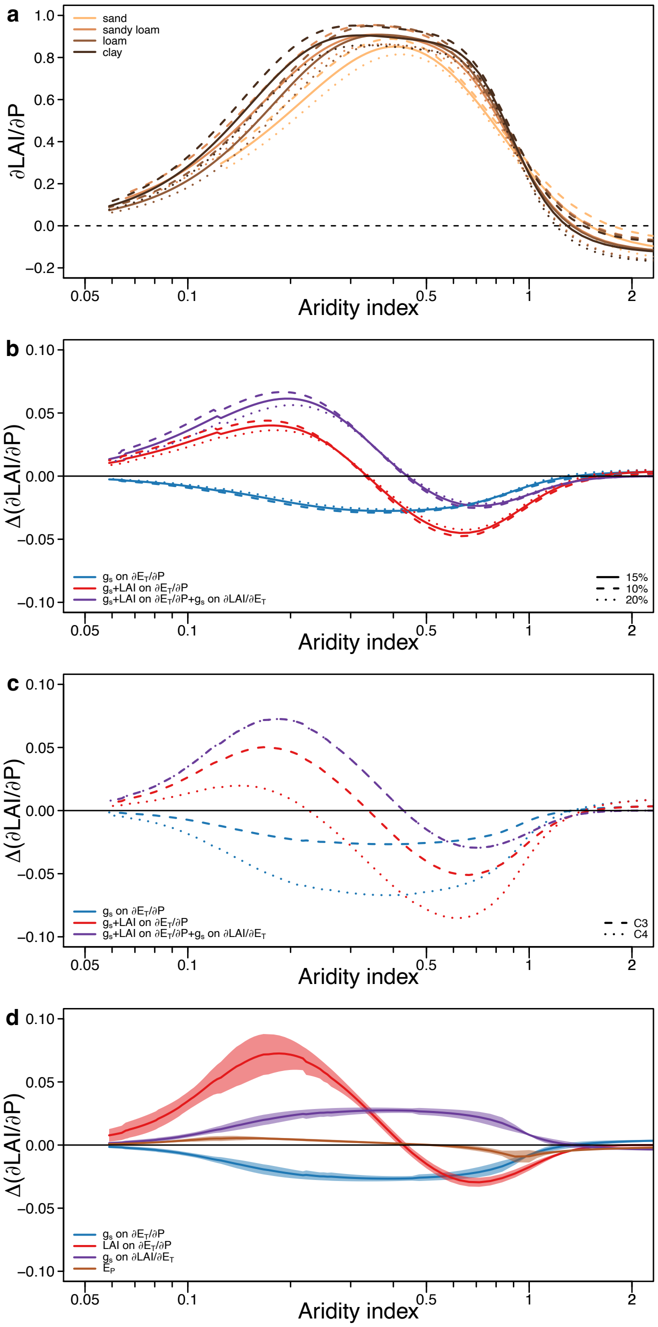

Supplementary Figure 18. Predicted responses of vegetation sensitivity to precipitation with different levels of interception and for C3 and C4. a vegetation sensitivity to precipitation predicted by the minimalistic model, with different line colors indicate soil types, and different line types for three levels of interception fraction (interception/precipitation). **b** changes in vegetation sensitivity to precipitation under different levels of interception fraction. **c** comparison between C3 and C4 plants in response to CO_2. **d** the effect of CO_2 on each individual factor for C3 plants. Solid lines and shades represent the mean and mean±std responses with different parameter combinations.

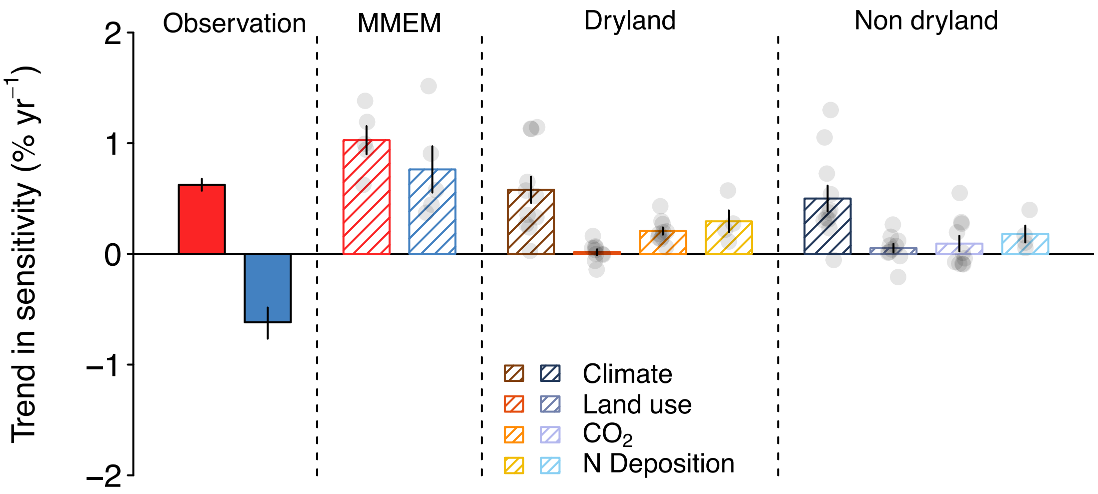

Supplementary Figure 19. Attribution of changes in precipitation sensitivity using MsTMIP model experiments. similar as Fig. 3, but only using GPP instead of LAI for the precipitation sensitivity calculation. The models are hatched since they are not comparable with observations from NDVI. The bars and error bars indicate the mean and standard error of the mean (SEM) of median trend across models, respectively. The sample sizes for each factor are: Observation 17601 and 14095 (for dryland and non-dryland, respectively); MMEM 5 and 5; Climate 11 and 11; Land use 10 and 10; CO_2 10 and 10; N Deposition 4 and 4.

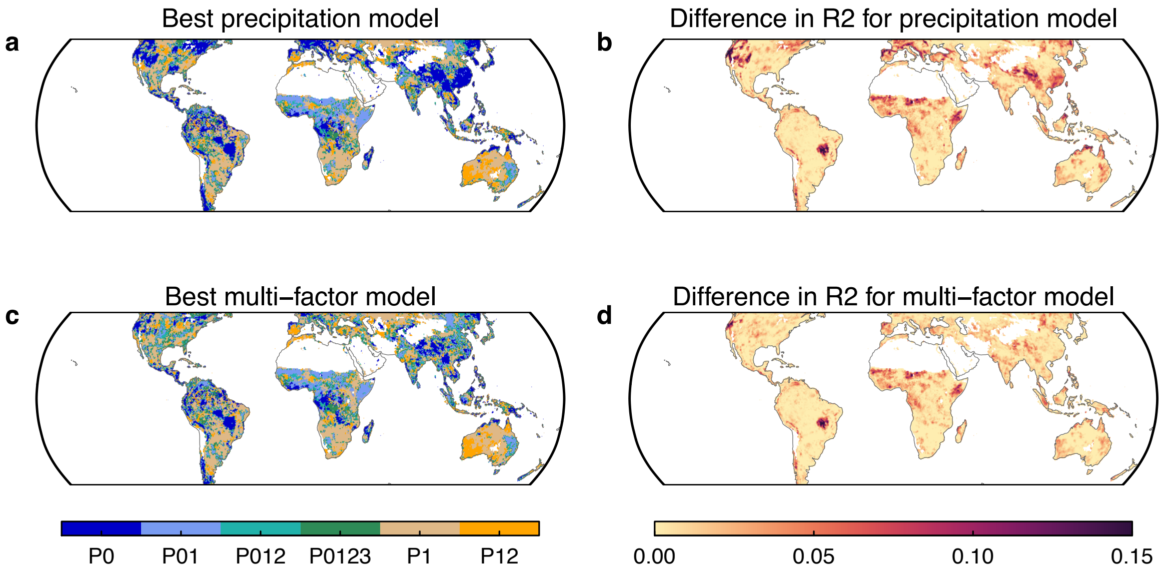

Supplementary Figure 20. The best model based on R^2 from multi linear regression. Comparison of model performance between different period to calculate precipitation for (**a**) univariate model and (**c**) multi-variate model. Numbers after P in the left legend indicate the months before current used for the multi linear regression. e.g., P0 indicate current month and P12 indicates average of precipitation from previous month and the month before. Difference in R^2 between the best model and the model using previous month precipitation (P1) for (**b**) univariate model and (**d**) multi-variate model.

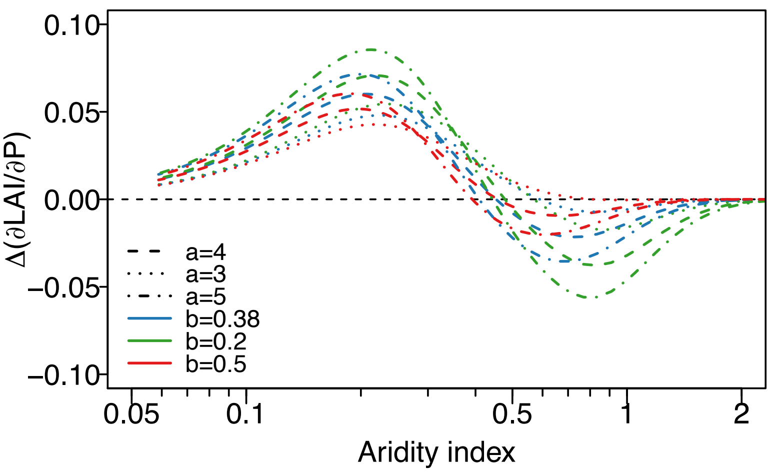

Supplementary Figure 21. Predicted responses of the changes in LAI to precipitation sensitivity along the aridity index. Different line types and colors correspond to different a and b combinations.

References

1. Wang, D., Wang, G. & Anagnostou, E. N. Evaluation of canopy interception schemes in land surface models. *Journal of Hydrology* **347**, 308–318 (2007).
2. Good, S. P., Moore, G. W. & Miralles, D. G. A mesic maximum in biological water use demarcates biome sensitivity to aridity shifts. *Nature Ecology & Evolution* **1**, 1883 (2017).
3. Medlyn, B. E. *et al.* Reconciling the optimal and empirical approaches to modelling stomatal conductance. *Global Change Biology* **17**, 2134–2144 (2011).
4. Walker, A. P. *et al.* Integrating the evidence for a terrestrial carbon sink caused by increasing atmospheric CO_2. *New Phytologist* (2020) doi:10.1111/nph.16866.
5. Donohue, R. J., Roderick, M. L., McVicar, T. R. & Yang, Y. A simple hypothesis of how leaf and canopy-level transpiration and assimilation respond to elevated CO_2 reveals distinct response patterns between disturbed and undisturbed vegetation: VEGETATION RESPONSES TO ELEVATED CO_2. *J. Geophys. Res. Biogeosci.* **122**, 168–184 (2017).
6. Ukkola, A. M. *et al.* Reduced streamflow in water-stressed climates consistent with CO2 effects on vegetation. *Nature Climate Change* **6**, 75–78 (2015).
7. Fatichi, S. *et al.* Partitioning direct and indirect effects reveals the response of water-limited ecosystems to elevated CO \textlesssub\textgreater2\textless/sub\textgreater. *Proceedings of the National Academy of Sciences* **113**, 12757–12762 (2016).
8. Porporato, A., Daly, E. & Rodriguez-Iturbe, I. Soil Water Balance and Ecosystem Response to Climate Change. *The American Naturalist* **164**, 625–632 (2004).
9. Wei, Y. *et al.* The North American Carbon Program Multi-scale Synthesis and Terrestrial Model Intercomparison Project – Part 2: Environmental driver data. *Geosci. Model Dev.* **7**, 2875–2893 (2014).
10. Thornton, P. E. *et al.* Modeling and measuring the effects of disturbance history and climate on carbon and water budgets in evergreen needleleaf forests. *Agricultural and Forest Meteorology* **113**, 185–222 (2002).
11. Huang, S. *et al.* Analysis of nitrogen controls on carbon and water exchanges in a conifer forest using the CLASS-CTEMN+ model. *Ecological Modelling* **222**, 3743–3760 (2011).
12. Oleson, K. W. *et al.* Technical Description of version 4.0 of the Community Land Model (CLM). *NCAR Technical Note* 266 (2010) doi:10.5065/D6RR1W7M.
13. Lei, H. *et al.* Sensitivity of global terrestrial gross primary production to hydrologic states simulated by the Community Land Model using two runoff parameterizations. *Journal of Advances in Modeling Earth Systems* **6**, 658–679 (2014).
14. King, A. W., Post, W. M. & Wullschleger, S. D. The Potential Response of Terrestrial Carbon Storage to Changes in Climate and Atmospheric CO2. *Climatic Change* **35**, 199–227 (1997).
15. Ricciuto, D. M., King, A. W., Dragoni, D. & Post, W. M. Parameter and prediction uncertainty in an optimized terrestrial carbon cycle model: Effects of constraining variables and data record length. *Journal of Geophysical Research: Biogeosciences* **116**, (2011).

16. Sitch, S. *et al.* Evaluation of ecosystem dynamics, plant geography and terrestrial carbon cycling in the LPJ dynamic global vegetation model. *Global Change Biology* **9**, 161–185 (2003).
17. Krinner, G. *et al.* A dynamic global vegetation model for studies of the coupled atmosphere-biosphere system: DVGM FOR COUPLED CLIMATE STUDIES. *Global Biogeochemical Cycles* **19**, (2005).
18. Schaefer, K. *et al.* Combined Simple Biosphere/Carnegie-Ames-Stanford Approach terrestrial carbon cycle model. *J. Geophys. Res.* **113**, G03034 (2008).
19. Zeng, N., Mariotti, A. & Wetzel, P. Terrestrial mechanisms of interannual CO_2 variability: INTERANNUAL CO_2 VARIABILITY. *Global Biogeochemical Cycles* **19**, (2005).
20. Ito, A. Changing ecophysiological processes and carbon budget in East Asian ecosystems under near-future changes in climate: implications for long-term monitoring from a process-based model. *J Plant Res* **123**, 577–588 (2010).
21. Yang, Y., Donohue, R. J. & McVicar, T. R. Global estimation of effective plant rooting depth: Implications for hydrological modeling. *Water Resour. Res.* **52**, 8260–8276 (2016).
22. Sheffield, J., Wood, E. F. & Roderick, M. L. Little change in global drought over the past 60 years. *Nature* **491**, 435–438 (2012).
