# Supplementary material for: Increasing sensitivity of dryland vegetation greenness to precipitation due to rising atmospheric CO2
Source: Nat Commun. 2022 Aug 19;13:4875. doi: 10.1038/s41467-022-32631-3 (PMC9391480; doi:10.1038/s41467-022-32631-3)
Supplement: Supplementary file 3 — Reporting Summary [file 41467_2022_32631_MOESM3_ESM.pdf]

nature portfolio

Corresponding author(s): Yao Zhang
Trevor F. Keenan

Last updated by author(s): Jul 31, 2022

# Reporting Summary

Nature Portfolio wishes to improve the reproducibility of the work that we publish. This form provides structure for consistency and transparency in reporting. For further information on Nature Portfolio policies, see our Editorial Policies and the Editorial Policy Checklist.

## Statistics

For all statistical analyses, confirm that the following items are present in the figure legend, table legend, main text, or Methods section.

| n/a | Confirmed | |
|---|---|---|
| ☐ | ☒ | The exact sample size ($n$) for each experimental group/condition, given as a discrete number and unit of measurement |
| ☒ | ☐ | A statement on whether measurements were taken from distinct samples or whether the same sample was measured repeatedly |
| ☐ | ☒ | The statistical test(s) used AND whether they are one- or two-sided<br>*Only common tests should be described solely by name; describe more complex techniques in the Methods section.* |
| ☒ | ☐ | A description of all covariates tested |
| ☒ | ☐ | A description of any assumptions or corrections, such as tests of normality and adjustment for multiple comparisons |
| ☐ | ☒ | A full description of the statistical parameters including central tendency (e.g. means) or other basic estimates (e.g. regression coefficient) AND variation (e.g. standard deviation) or associated estimates of uncertainty (e.g. confidence intervals) |
| ☐ | ☒ | For null hypothesis testing, the test statistic (e.g. $F$, $t$, $r$) with confidence intervals, effect sizes, degrees of freedom and $P$ value noted<br>*Give P values as exact values whenever suitable.* |
| ☐ | ☒ | For Bayesian analysis, information on the choice of priors and Markov chain Monte Carlo settings |
| ☒ | ☐ | For hierarchical and complex designs, identification of the appropriate level for tests and full reporting of outcomes |
| ☐ | ☒ | Estimates of effect sizes (e.g. Cohen's $d$, Pearson's $r$), indicating how they were calculated |

*Our web collection on statistics for biologists contains articles on many of the points above.*

## Software and code

Policy information about availability of computer code

| | |
|---|---|
| Data collection | No software was used to collect data. |
| Data analysis | Data analysis were performed using R 3.5.2 and Python 3.7.0. The code for the analysis is available at https://github.com/zhangyaonju/prec_sensitivity/. Details were reported in the Method section. |

For manuscripts utilizing custom algorithms or software that are central to the research but not yet described in published literature, software must be made available to editors and reviewers. We strongly encourage code deposition in a community repository (e.g. GitHub). See the Nature Portfolio guidelines for submitting code & software for further information.

## Data

Policy information about availability of data

All manuscripts must include a data availability statement. This statement should provide the following information, where applicable:

- Accession codes, unique identifiers, or web links for publicly available datasets
- A description of any restrictions on data availability
- For clinical datasets or third party data, please ensure that the statement adheres to our policy

All data used in the analysis are publicly accessible. The GIMMS NDVI 3g v1 dataset is available at http://poles.tpdc.ac.cn/en/data/9775f2b4-7370-4e5e-a537-3482c9a83d88/, the CRU climate dataset is available at https://crudata.uea.ac.uk/cru/data/hrg/, the GPCC precipitation data is available at https://www.dwd.de/EN/ourservices/gpcc/gpcc.html, the MsTMIP model outputs are available at https://doi.org/10.3334/ORNLDAAC/1225, the CRU-NCEP V6 dataset is available through https://doi.org/10.3334/ORNLDAAC/1220, The aridity index data is available at https://doi.org/10.6084/m9.figshare.7504448.v3

## Human research participants

Policy information about studies involving human research participants and Sex and Gender in Research.

| | |
|---|---|
| Reporting on sex and gender | NA |
| Population characteristics | NA |
| Recruitment | NA |
| Ethics oversight | NA |

Note that full information on the approval of the study protocol must also be provided in the manuscript.

# Field-specific reporting

Please select the one below that is the best fit for your research. If you are not sure, read the appropriate sections before making your selection.

☐ Life sciences ☐ Behavioural & social sciences ☒ Ecological, evolutionary & environmental sciences

For a reference copy of the document with all sections, see nature.com/documents/nr-reporting-summary-flat.pdf

# Ecological, evolutionary & environmental sciences study design

All studies must disclose on these points even when the disclosure is negative.

| | |
|---|---|
| Study description | This study presents a satellite estimated long term increase of vegetation sensitivity to precipitation in global drylands. This robust trend is corroborated by model simulations and hydrological theory, both suggest that elevated atmospheric CO2 contributes to this increasing trend of vegetation sensitivity to precipitation in global drylands. |
| Research sample | We used satellite derived GIMMS NDVI and leaf area index, gross primary production, transpiration, evapotranspiration simulations from 10 models that participated the MsTMIP project. We also used various climate datasets to test the robustness of the precipitation sensitivity calculation. These data are available through the links described in the Data section. The observations/simulations are spatially contiguous and can represent the entire study region. |
| Sampling strategy | We used satellite data and model simulations for vegetated land in low-to-mid latitude. To get the average response of the precipitation sensitivity along aridity index, we used bootstrap method and calculated the median of the sensitivity. |
| Data collection | All datasets were downloaded from Interenet using the URLs in the data availability statement in the main text. The NDVI data is from a series of NOAA satellites. CRU climate and GPCC precipitation datasets are interpolated from weather station observations. The MsTMIP model outputs are from computer simulations. The aridity index is derived from gridded climate data obtained from weather stations and spatial modeling. |
| Timing and spatial scale | The GIMMS NDVI 3g dataset has a 15-day temporal and 0.083 degree spatial resolution and is available during 1981-2015. The model simulations from MsTMIP have a monthly temporal resolution and 0.5 degree spatial resolution, covering 1980-2011. The spatial extent of our study is the land area between 50 degree North and 50 degree South. The time period we chose is restricted to the availability of satellite data. The satellite observations and model simulations are continuously through time during the study period. |
| Data exclusions | We excluded three models which present obvious errors in the precipitation sensitivity calculation. |
| Reproducibility | Our analyses were based on public satellite datasets, terrestrial biome model outputs, climate products and well-defined methods, the results could be reliably reproduced. |

| | |
|---|---|
| Randomization | Randomization does not apply in this study as we performed wall-to-wall mapping globally with satellite images and gridded climate dataset. |
| Blinding | Our study only used existing global gridded datasets which do not involve sample collection or selection, therefore blinding is not relevant to our study. |

Did the study involve field work? ☐ Yes ☒ No

# Reporting for specific materials, systems and methods

We require information from authors about some types of materials, experimental systems and methods used in many studies. Here, indicate whether each material, system or method listed is relevant to your study. If you are not sure if a list item applies to your research, read the appropriate section before selecting a response.

## Materials & experimental systems

| n/a | Involved in the study |
|---|---|
| ☒ | ☐ Antibodies |
| ☒ | ☐ Eukaryotic cell lines |
| ☒ | ☐ Palaeontology and archaeology |
| ☒ | ☐ Animals and other organisms |
| ☒ | ☐ Clinical data |
| ☒ | ☐ Dual use research of concern |

## Methods

| n/a | Involved in the study |
|---|---|
| ☒ | ☐ ChIP-seq |
| ☒ | ☐ Flow cytometry |
| ☒ | ☐ MRI-based neuroimaging |
